# Intra-articular injection of decellularized extracellular matrices in the treatment of osteoarthritis in rabbits

**DOI:** 10.7717/peerj.8972

**Published:** 2020-04-22

**Authors:** Yaxin Zhang, Jihang Dai, Lianqi Yan, Yu Sun

**Affiliations:** 1 Dalian Medical University, Dalian, China; 2 Northern Jiangsu People’s Hospital, Yangzhou, China

**Keywords:** Decellularization, ECM (extracellular matrix), Articular cartilage, Particulated cartilage, Osteoarthritis

## Abstract

**Background:**

We investigated the role of decellularized cartilage matrix in osteoarthritis to seek a new treatment for this disease.

**Methods:**

Knee cartilage from rabbits was decellularized and the degree of decellularization was assessed. A grinder was used to turn acellular cartilage into particles, which were then used in a suspension. Thirty New Zealand white rabbits were subjected to an operation on their anterior cruciate ligament for the osteoarthritis model. The success of the animal model of osteoarthritis was evaluated using results from six rabbits. The remaining 24 rabbits were randomly divided into four groups (groups A, B, C, and D). Rabbits in groups A, B, C, and D were injected with 200 µl of normal saline, 200 µl of 10% (w/v) cartilage decellularized suspension, 200 µl of 20% (w/v) cartilage decellularized suspension, and 200 µl of 40% (w/v) cartilage decellularized suspension into the knee joints, respectively. Macroscopic and microscopic assessments were performed three months after surgery to assess the degree of osteoarthritic changes.

**Results:**

Histological and biochemical analysis revealed that the cartilage decellularized matrix removed cells after decellularization but retained components of collagen and glycosaminoglycan. Group A exhibited the most significant changes from osteophyte and cartilage erosion, which was macroscopically observable on the surface of the femoral cartilage. HE staining in group A revealed damage to the cartilage surface, disorganized chondrocytes, and spontaneous fibrocartilage formation. Safranin O-fast green staining revealed a cavity formed at the osteochondral junction in group A that did not appear in other groups.

**Conclusion:**

Our study shows that decellularized cartilage matrix has a certain therapeutic effect on osteoarthritis and provides new insights in the treatment of osteoarthritis.

## Introduction

Osteoarthritis (OA) is a degenerative joint disease characterized by the destruction of the articular cartilage, causing chronic pain and the loss of joint function. OA is the most common factor for disability among elderly adults, occurring in 10 percent of those over the age of 60. Elderly adults with OA face considerable economic and medical burdens and typically require healthcare support from the government (Jacer, Shafaei & Soleimani Rad, 2018; Peat, McCarney & Croft, 2001). OA currently has no effective treatments outside of pain management and the administration of supplements. OA is a progressive disease and joint replacement surgery is required when drug therapy cannot relieve joint pain (Shah et al., 2018). However, joint replacement surgery is costly and the artificial joint has a limited lifetime, creating an urgent need to find a treatment that can delay the progression of OA.

The extracellular matrix (ECM) is a composite of the secreted products of resident cells. It contains biologically active substances associated with cell proliferation, migration, and adhesion, which are unique to individual tissues (Badylak, 2004). Physical and chemical methods are used to remove cells from tissues to obtain decellularized extracellular matrices (dECM), reducing their antigenicity (Xue et al., 2018; Zhang et al., 2017). dECM have a relatively low immunogenicity due to the removal of cellular components, allowing them to be implanted in allogeneic bodies (Kang et al., 2014). A variety of decellularized tissues have been approved for use in humans, including the human dermis, porcine small intestinal submucosa, porcine heart valves, and porcine bladders (Gilbert, Sellaro & Badylak, 2006). Studies have shown that dECM may be beneficial to damaged tissue because they retain most of their original structure and protein content (Badylak, 2007). dECM can promote the differentiation of embryonic stem cells into specific cells and cartilage extract can promote the differentiation of embryonic stem cells into cartilage in vitro (Jin et al., 2007). The acellular matrix of cartilage is mainly used for cartilage defect repairs and to functionalize other biological materials to enhance cartilage formation. However, there are few studies on the application of the acellular matrix of cartilage in OA (Almeida et al., 2016; Sutherland & Detamore, 2015; *Visser et al., 2015*).

We injected cartilage-derived dECM into the knee joint of rabbits with OA and assessed the knee repair using macroscopic and histological outcomes. We sought to determine the potential application of dECM for the treatment of OA.

## Materials & Methods

### Decellularization

Six fresh rabbit knee joints were obtained from experimental rabbits in a medical laboratory (Yangzhou University, Yangzhou, China). The joints were transported on ice to our laboratory within 2 h of collection. A blade was used to obtain cartilage from the femoral side of the articular surface after the joint was dissected. The cartilage pieces were washed thoroughly in phosphate-buffered saline (PBS) at 4 °C to remove blood and impurities.

We used the decellularization process based on the method by Kheir with some improvements (*Kheir et al., 2011)*. The collected cartilage pieces were frozen at −40 °C for 12 h and were left on the experimental bench to thaw at room temperature for 3 h; this process was performed twice. The specimens were then placed in a hypotonic solution (10 mM Tris-HCl, pH = 8.0) (Solarbio, Beijing, China) for another two freeze-thaw cycles, as mentioned above. The specimens were then placed in a hypotonic buffer (10 mM Tris-HCl, pH=8.0) for 24 h at 45 °C before being washed with an ionic detergent (0.1% (w/v) sodium dodecyl sulfate (SDS) (Solarbio, Beijing, China), 10 KIU/ml aprotinin (Sigma Aldrich, America), 0.1% (w/v) ethylene diamine tetraacetic acid (EDTA) (Solarbio, Beijing, China), and 10 mM Tris-HCl) at 45 °C for 24 h. This hypotonic/SDS cycle was repeated three times. The specimens were then immersed in PBS with aprotinin (10 KIU/ml) for 30 min and were left to stand for 24 h at 45 °C; this process was performed twice. The specimens were then placed in a nuclease solution (50 mM Tris-HCl solution (pH 7.5), 10 mM magnesium chloride (Solarbio, Beijing, China), 50 µg/mL of bovine serum albumin (BSA) (Solarbio, Beijing, China), 50 U/ml DNase (Sigma Aldrich, America), and 1 U/ml RNase (Sigma Aldrich, America)) at 37 °C for 3 h. The specimens were soaked in PBS with aprotinin (10 KIU/ml) for another 24 h at 45 °C and placed in PBS with 0.1% (w/v) Triton X-100 (Sigma Aldrich, America) for 24 h at 45 °C. Finally, the specimens were immersed and washed in PBS for 24 h at 45 °C.

100 U/ml penicillin (Solarbio, Beijing, China), 100 µg/ml streptomycin (Solarbio, Beijing, China), and 2.5 µg/ml fluconazole (Solarbio, Beijing, China) were added to all of our solutions and the specimens were placed under ultraviolet light for disinfection.

### Decellularization evaluation

#### Histology

All of the cartilage pieces were placed in 4% (w/v) paraformaldehyde at room temperature for 24 h before being incubated in a decalcifying solution of 25% (w/v) EDTA solution (pH 7.0). The liquid was changed every 3 days until the cartilage was soft enough to be sectioned. A hematoxylin-eosin (H&E) stain was used to visualize the cellular contents. DAPI (4′,6-diamidino-2-phenylindole) (Solarbio, Beijing, China) staining was used to visualize deoxyribonucleic acid (DNA), toluidine blue staining was used to visualize glycosaminoglycan (GAG), and immunohistochemistry was applied to characterize the changes in type II collagen.

#### Biochemical assays

The GAG and DNA content of the samples were quantified using the method previously reported (Bolland et al., 2007). Fresh cartilage pieces and acellular cartilage pieces were digested in a papain solution (125 mg/mL papain, five mm cysteine HCl, and five mm EDTA in PBS) at 60 °C for 12 h. The resulting solution was centrifuged at 10,000 g for 30 min and stored at −80 °C before testing.

#### Glycosaminoglycan assay

The GAG content was evaluated using a dimethylmethylene blue colorimetric quantitative detection kit (Jianglai, Shanghai, China). 50 µl of the papain digestion solution with cartilage pieces was mixed with 1 ml 1, 9-dimethylmethylene blue dye reagent by shaking for 15 s. The mixture was then incubated for 30 min at room temperature away from light and was shocked for 15 s every 5 min. The solutions were centrifuged at 16,000 g for 10 min after the incubation period. The liquid was carefully aspirated and the remaining precipitate was dissolved in 1 ml of a dissociation solution and incubated for 5 min at room temperature in the dark. The absorbance was read in the spectrophotometer at 656 nm. The GAG content was calculated by the interpolation from a standard curve generated using standard samples.

#### Deoxyribonucleic acid assay

DNA content was evaluated using the dsDNA HS Assay Kit for Qubit (Yeasen, Shanghai, China). 10 µl of the papain digestion solution made from cartilage slices was mixed with 190 µl of the test solution. The fluorescence value of the sample was measured using a fluorometer after being incubated for 2 min in the dark. The DNA content was calculated from a standard curve generated from standard samples.

#### Microstructure

The fresh cartilage pieces and acellular cartilage pieces were immersed in 2.5% (w/v) glutaraldehyde in a PBS solution for 2 h before being washed with a sodium dimethylarsenate buffer (pH 7.4) and fixed with 1% (w/v) osmium tetroxide. The cartilage pieces were coated with gold-palladium and scanned with a scanning electronic microscope (SEM) (SU8100, HITACHI, Japan). Pore size and porosity were measured using the mercury intrusion method.

#### Manufacturing of decellularized extracellular matrix (dECM) suspensions

Processed cartilage pieces were ground into particle using a grinder, and their diameter was measured using Alcian blue staining (Solarbio, Beijing, China). The dECM particles were washed thoroughly with normal saline and suspensions were created using the particles at 10%, 20%, and 40% concentrations (w/v).

### Animal experiment

#### Induction of rabbit osteoarthritis (OA)

Thirty-six male New Zealand rabbits, weighing 2–2.5 kg each, were used for this experiment. This study was approved by the Animal Research Committee of Yangzhou University and all animal testing followed the Guide for the Care and Use of Laboratory Animal developed by the National Academies. The rabbits were anesthetized with 1% (w/v) sodium pentobarbital (3 ml/kg) through their ear veins before an incision was made in the knee joint. The soft tissue was bluntly separated to dislocate the tibia and expose the femoral condyle. Thirty rabbits were randomly assigned to have the anterior cruciate ligament (ACL) cut off and the response of the rabbits to this excision was tested using anterior drawer test; these rabbits were known as the experimental group. The remaining six rabbits had their knee joints surgically exposed but did not have the ACL cut off and were referred to as the control group. The knee joints of both the experimental and control animals were sutured in layers with absorbable silk sutures. Cefazolin (Huarun, Shenzhen, China) was administered 30 min before surgery and 3 days after surgery through an intramuscular injection to prevent infection. Celecoxib (Pfizer, America) was administered for 1 week after surgery to relieve pain. It took 6 weeks to induce a rabbit knee OA model.

#### Evaluation of the OA model

Six experimental rabbits whose ACLs were cut and six control rabbits who retained their ACL were randomly selected six weeks after the induction of OA. The rabbits were anesthetized with 1% (w/v) sodium pentobarbital (3 ml/kg) through their ear veins. Magnetic resonance imaging (MRI) was used to scan the knee joints, after which the knee joints were opened to observe the macroscopic morphology.

#### Intra-articular injection dECM suspension

The remaining rabbits from the experimental group were randomly divided into four groups (A, B, C, and D). Rabbits in groups A, B, C, and D were injected with 200 µl of normal saline, 200 µl of 10% dECM suspension, 200 µl of 20% dECM suspension, and 200 µl of 40% dECM suspension into the knee joints, respectively.

#### General observation

Rabbits were euthanized 3 months after the intra-articular injection of the dECM suspension. The knee joints were dissected and the cartilage of the femoral condyle was observed.

#### Histological observation

The femoral side of the knee was removed and placed in 4% (w/v) paraformaldehyde for 48 h. The femoral side of the knee was then incubated in a decalcifying solution of 25% (v/w) EDTA solution (pH 7.0) at room temperature. The liquid was changed every 3 days until the femoral side of the knee was soft enough to be sectioned. The treated tissues were embedded in paraffin and sliced. The histological sections were stained with H&E and safranin O-fast green staining.

### Statistical analysis

The Statistical Package for the Social Sciences version 19.0 statistical software was used to analyze the experimental data. All data were expressed as mean ± standard deviation. A *p*-value of <0.05 was considered to be statistically significant.

## Results

### Decellularization evaluation

#### Histological observation

**Figure 1:**
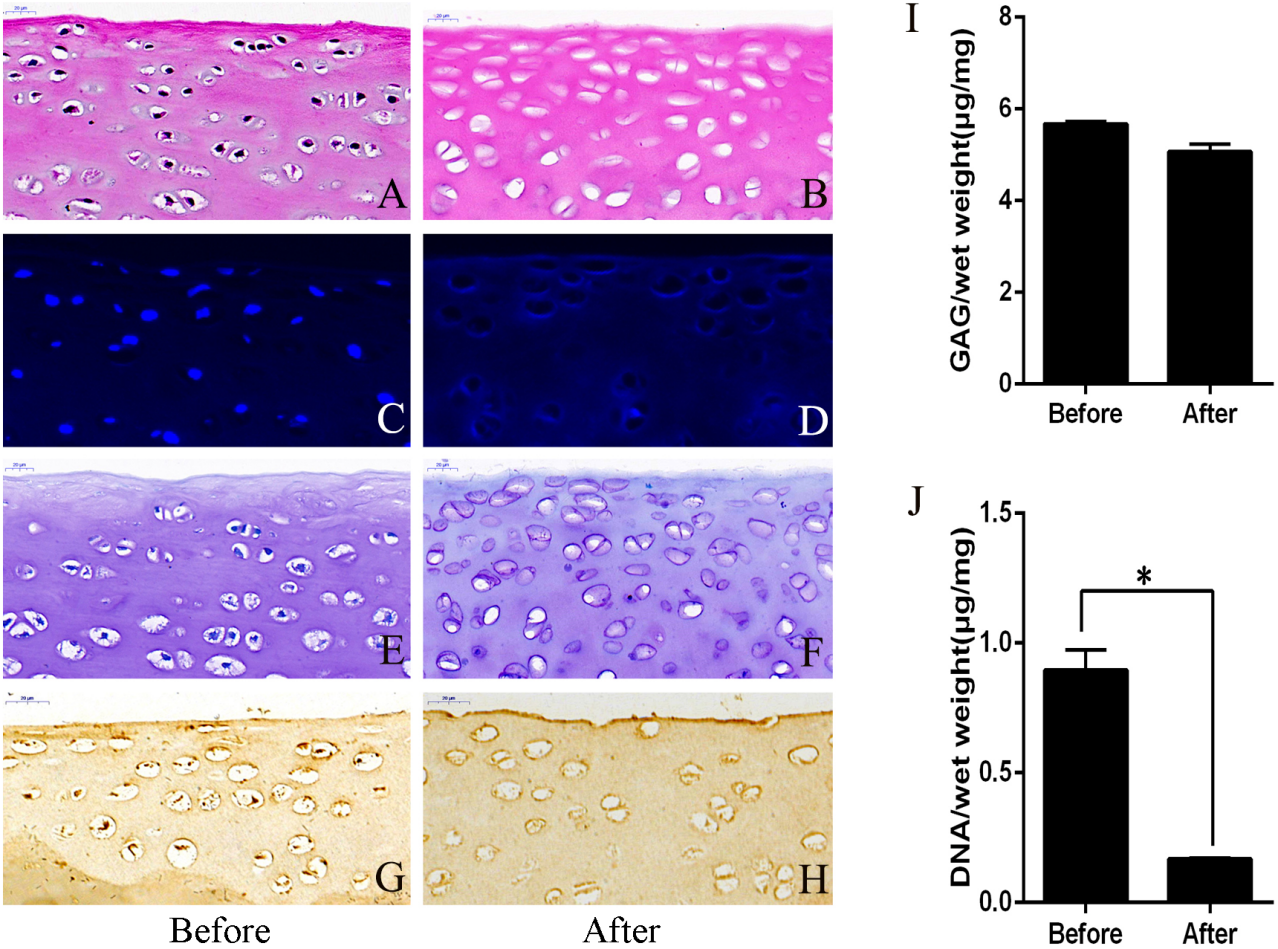
Evaluation of cartilage before and after decellularization procedure. (A and B) Hematoxylin-eosin staining revealed that the nucleus was almost completely removed after decellularization. (C and D) DAPI staining showed that the DNA was almost completely removed after decellularization. (E and F) Toluidine blue staining showed no significant change in GAG after decellularization. (G and H) Immunohistochemistry showed no significant change in type II collagen after decellularization. GAG content (I) and DNA content (J). ∗*p* < 0.05. GAG, glycosaminoglycan.

The decellularization protocol resulted in cartilage pieces similar in size and appearance to native tissues with a near-complete removal of the cellular nuclei (Figs. 1A and 1B). There was a similar GAG content between the decellularized and the un-decellularized cartilage as shown with toluidine blue staining (Figs. 1E and 1F). DNA in the dECM was not observed under DAPI staining when compared to the un-decellularized cartilage (Figs. 1C and 1D). Immunohistochemical staining for type II collagen was positive and the intensity of the staining was similar for both between the decellularized and the un-decellularized cartilage (Figs. 1G and 1H).

#### Biochemical analysis

The GAG content of the cartilage pieces before and after decellularization per mg wet weight were 5.68 ± 0.53 µg/mg and 5.07 ± 0.16 µg/mg, and the amounts of DNA per mg wet weight of the cartilage pieces were 0.90 ± 0.08 µg/mg and 0.17 ± 0.02 µg/mg, respectively. There was no significant difference (*P* > 0.05) in GAG content before and after decellularization (Fig. 1I). DNA content was significantly lower (*P* < 0.05) after decellularization than before decellularization (Fig. 1J).

#### Microstructural analysis

**Figure 2:**
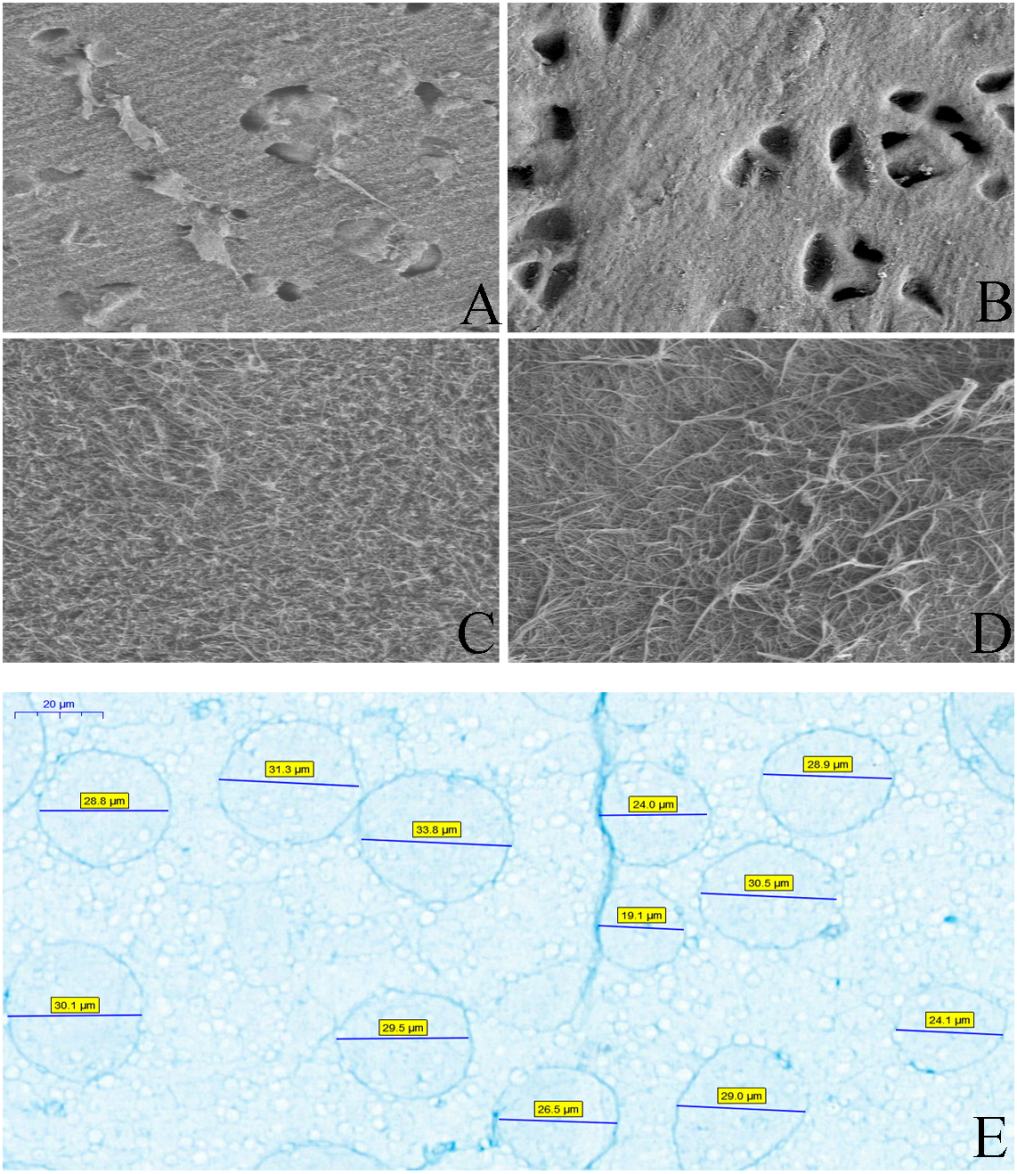
The scanning electron microscope results of cartilage and the evaluation of particles. (A) A large number of cells in the pores before decellularization (original magnification x500). (B) There were no more cells in the pores after the decellularization (original magnification x500). (C) The cartilage surface tissue was dense before decellularization (original magnification ×2000). (D) The cartilage surface tissue becomes loose after decellularization (original magnification ×2000). (E) The size of the cartilage acellular matrix particles was measured by Alcian Blue staining.

SEM analysis demonstrated that decellularized cartilage lost many cells and that the surface structure was not as dense as the un-decellularized cartilage (Fig. 2). Mercury intrusion porosimetry was used to determine that the pore size was larger with a greater pore density in the decellularized cartilage piece than in the un-decellularized cartilage piece (7.57 µm vs 7.25 µm and 1615.19 n/mm^2^ vs 994.93 n/mm^2^, respectively).

#### Characterizing the dECM particle

dECM particles were stained with Alcian blue and the particle size was analyzed using Image J and was determined to be 27.97  ± 3.96 µm (Fig. 2E).

### OA model evaluation

**Figure 3:**
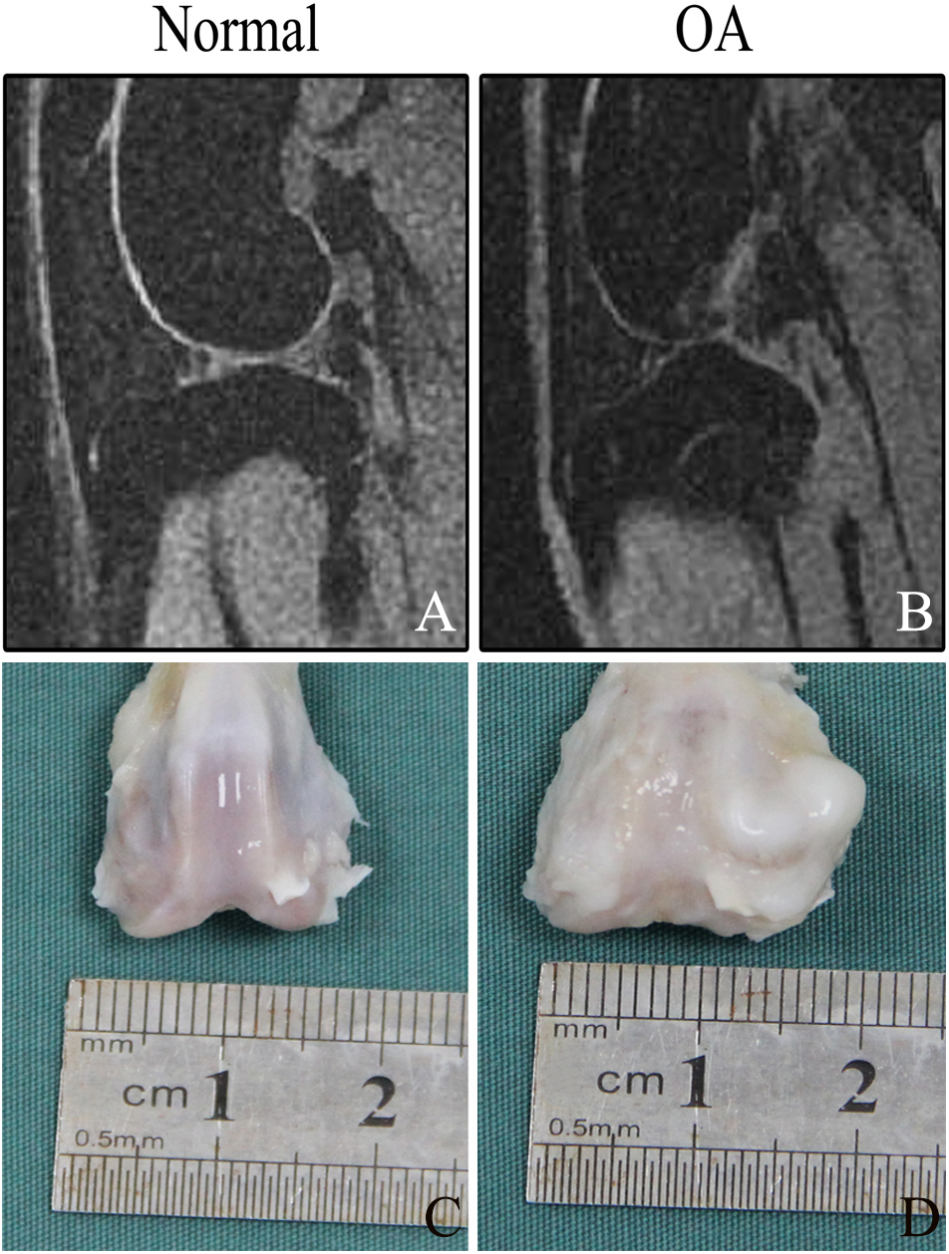
Evaluation of osteoarthritis model. (A) The cartilage continuity was intact under magnetic resonance imaging, and no obvious damage was seen. (B) The continuous destruction of the cartilage and the thinning of the cartilage layer were observed under magnetic resonance imaging. (C) The cartilage surface was smooth with no obvious damage. (D) The cartilage surface shows erosion and the integrity of the surface cartilage was disrupted.

The cartilage surfaces from the experimental rabbits showed erosion of the cartilage tissues with poorer integrity than the control group (Figs. 3C and 3D). Callus were observed on the femoral side of the knee and continuous destruction of the cartilage and the thinning of the cartilage layer were observed using MRI (Figs. 3A and 3B).

These results confirmed that the OA model was successful.

### Macroscopic findings

**Figure 4:**
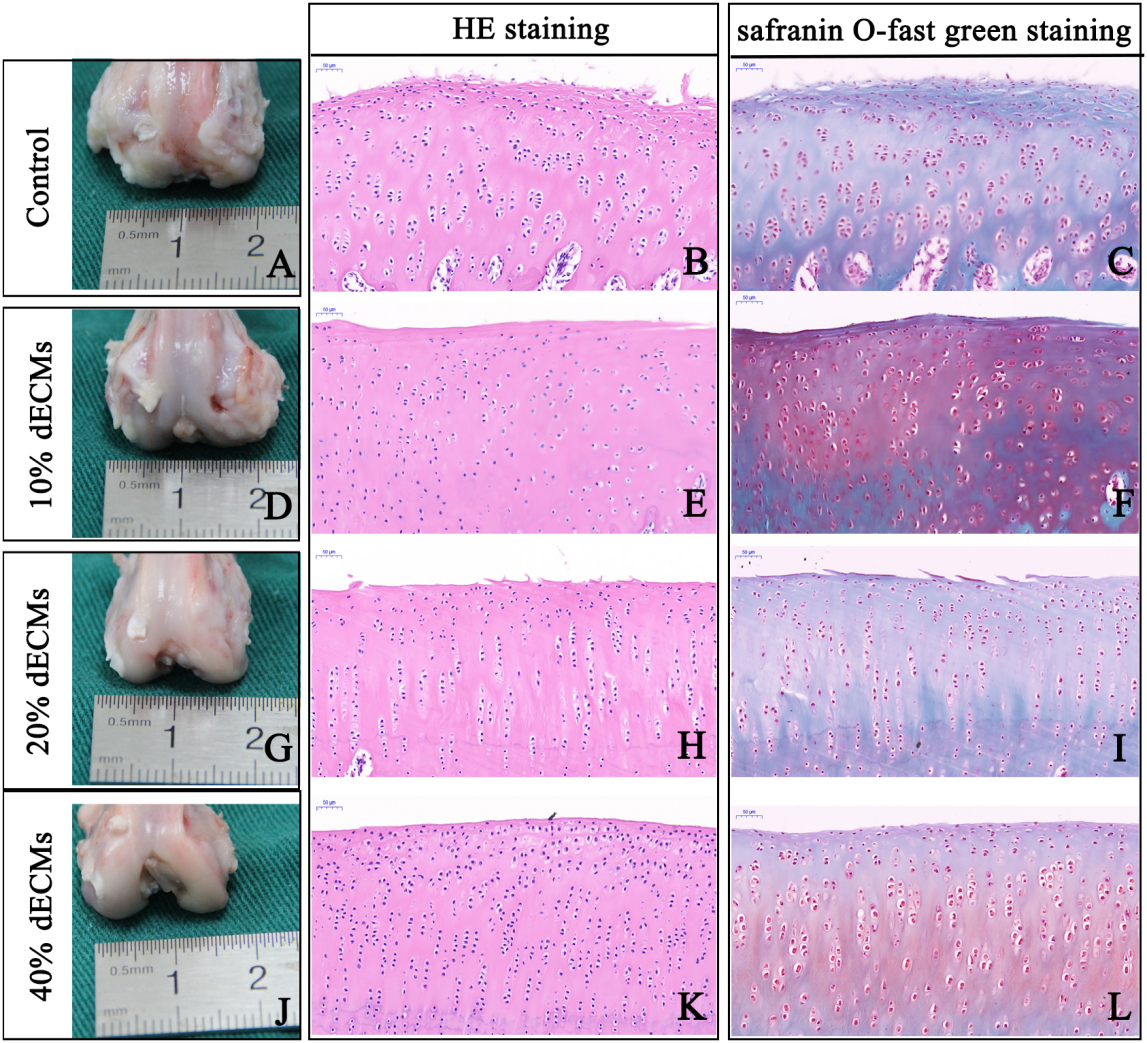
Representative macrographs and micrographs of knee joint samples. In the control group: the surface of the cartilage was damaged, the chondrocytes were arranged disorderly and the bone-cartilage junction formed a cavity (A–C). In the 10% (w/v) acellular cartilage suspension group, the cartilage surface was layered, the chondrocytes were arranged disorderly and the bone-cartilage were connected intact (D–F). In the 20% (w/v) acellular cartilage suspension group, the cartilage surface was not smooth, the chondrocytes were neat and the osteochondral connection was intact, but the number of chondrocytes were reduced (G–I). In the 40% (w/v) acellular cartilage suspension group, the cartilage surface was smooth, the chondrocytes were neat and the osteochondral connection was intact (J–L).

All rabbits survived the experimental period with no signs of major wound infections and were euthanized twelve weeks after surgery. We observed significantly fewer osteophytes and instances of cartilage erosion on the cartilage surface of the femoral side of the knee joint in groups B, C, and D compared to group A (Figs. 4A, 4D, 4G and 4J).

### Microscopic findings

The degree of OA was evaluated using H&E and safranin O-fast green staining related to the smoothness of the articular cartilage surface, the distribution of chondrocytes, and the integrality of osteochondral junction. In group A, the surface of the articular cartilage was destroyed. The chondrocytes were disorganized, a cavity had formed between the connection of the cartilage and bone, and fibrocartilage was formed on the surface of the cartilage (Figs. 4B and 4C). In group B, the surface of the articular cartilage was layered with disorganized chondrocytes and there was an intact osteochondral connection (Figs. 4E and 4F). In group C, the surface of the articular cartilage was not smooth but there was a normal distribution of chondrocytes and an intact osteochondral connection. However, the number of chondrocytes was reduced (Figs. 4H and 4I). In group D, the surface of the articular cartilage was smooth with a normal distribution of chondrocytes and an intact osteochondral connection (Figs. 4K and 4L).

## Discussion

Our findings are the first to indicate that dECM derived from cartilage have a positive effect on the treatment of knee OA in rabbits, which may lead to a potential treatment for OA of the knee.

The degeneration of articular cartilage is the major pathological feature of osteoarthritis. The articular cartilage contains a single chondrocyte, which makes it difficult for the articular cartilage to repair itself after an injury due to insufficient articular cartilage blood vessels and nerve innervations *(Madeira et al., 2015; Smith, Knutsen & Richardson, 2005)*. The present treatment of knee OA mainly focuses on symptomatic treatment due to a lack of effective methods to treat the disease *(Kurose et al., 2010*). Joint cavity injections are less invasive and less costly to patients, making them an attractive treatment option for many patients. Sodium hyaluronate and hormones are typically used in intra-articular injections. Sodium hyaluronate mainly functions as a joint lubricant and cannot stop or reverse the pathological process of OA (Thomas et al., 2013). There are adverse effects of hormonal drugs on the cartilage, limiting its clinical application. Therefore, it is necessary to develop drugs that can be injected locally into the joint that delay articular cartilage degeneration and have a curative effect for the treatment of OA.

dECM was studied in the early 1990s with the intent of removing the immunogenicity of the tissue to create a natural tissue substitute without the risk of immune rejection (Heath, 2019). Decellularized dermal matrices (Alloderm) have been approved by the Food Drug Administration for clinical application since that time (Jansen et al., 2013). Acellular treatment of the dermis, pericardium, cornea, blood vessel, bladder, esophagus, small intestine mucosal matrix, bone, liver, and other substitutes for corresponding tissues has been successfully developed both in animal experiments and clinical applications. dECM is shown to have good clinical application (Iorio, Shuck & Attinger, 2012). dECM is derived from acellular components of tissues and organs and are ECM derivatives. The materials have low immunogenicity due to the removal of cellular components yet they retain the three-dimensional structure of natural tissue and other important components of the ECM, such as bioactive factors, which can promote cell adhesion, migration and proliferation. Collagen, non-collagen glycoprotein, elastin, and GAG of the ECM have significantly important biological functions and can affect cell shape and control cell migration, proliferation, differentiation, and metabolism. Experiments have shown that dECM could induce epithelial and smooth muscle cell growth and when it is applied in vivo it may grow into a part of the body (Badylak et al., 2001; Holton et al., 2007; Urita et al., 2007).

More than 90% of the normal cartilage tissue consists of type II collagen and mucopolysaccharides (GAG). A small number of chondrocytes maintain the balance of cartilage metabolism by secreting a variety of factors (Welton et al., 2018). According to previous studies, cartilage-derived dECM was generally made into materials to promote chondrocyte or stem cell growth to treat cartilage defects. Previous in vitro studies have shown that dECM derived from cartilage has a positive effect on ECM secretion by chondrocytes and can effectively stimulate the chondrocyte to produce ECM (Pei et al., 2010). In this experiment, we obtained decellularized cartilage pieces of allogeneic knee cartilage using physical and chemical methods and ground the samples into particles using a grinder. dECM particles and normal saline were prepared into solvents with different wet-weight ratios based on a previous study of a minimally invasive treatment using dECM derived from the cartilage. The prepared suspension was injected into the knee joint of rabbits with OA to produce a positive effect on knee OA. Our results showed that this treatment can effectively alleviate the progression of OA without serious complications, including infection and skin necrosis.

## Conclusions

We investigated the therapeutic effects of a decellularized cartilage matrix on a rabbit OA model. Our results showed that this treatment positively affects the outcome of knee OA. However, our experiment was limited. There is no way to trace the dECM particles injected into the knee joint so there is no way to know where they settled. In summary, we investigated the therapeutic effect of dECM derived from the cartilage on knee OA to provide potential clinical treatment options for OA of the knee.

##  Supplemental Information

10.7717/peerj.8972/supp-1 Supplemental Information 1 Raw data

## Additional Information and Declarations

### Data Availability

The following information was supplied regarding data availability: The raw measurements available in the Supplementary Files.
