# Supplementary figures and images for: Intra-articular injection of decellularized extracellular matrices in the treatment of osteoarthritis in rabbits
Source: PeerJ. 2020 Apr 22;8:e8972. doi: 10.7717/peerj.8972 (PMC7183306; doi:10.7717/peerj.8972)

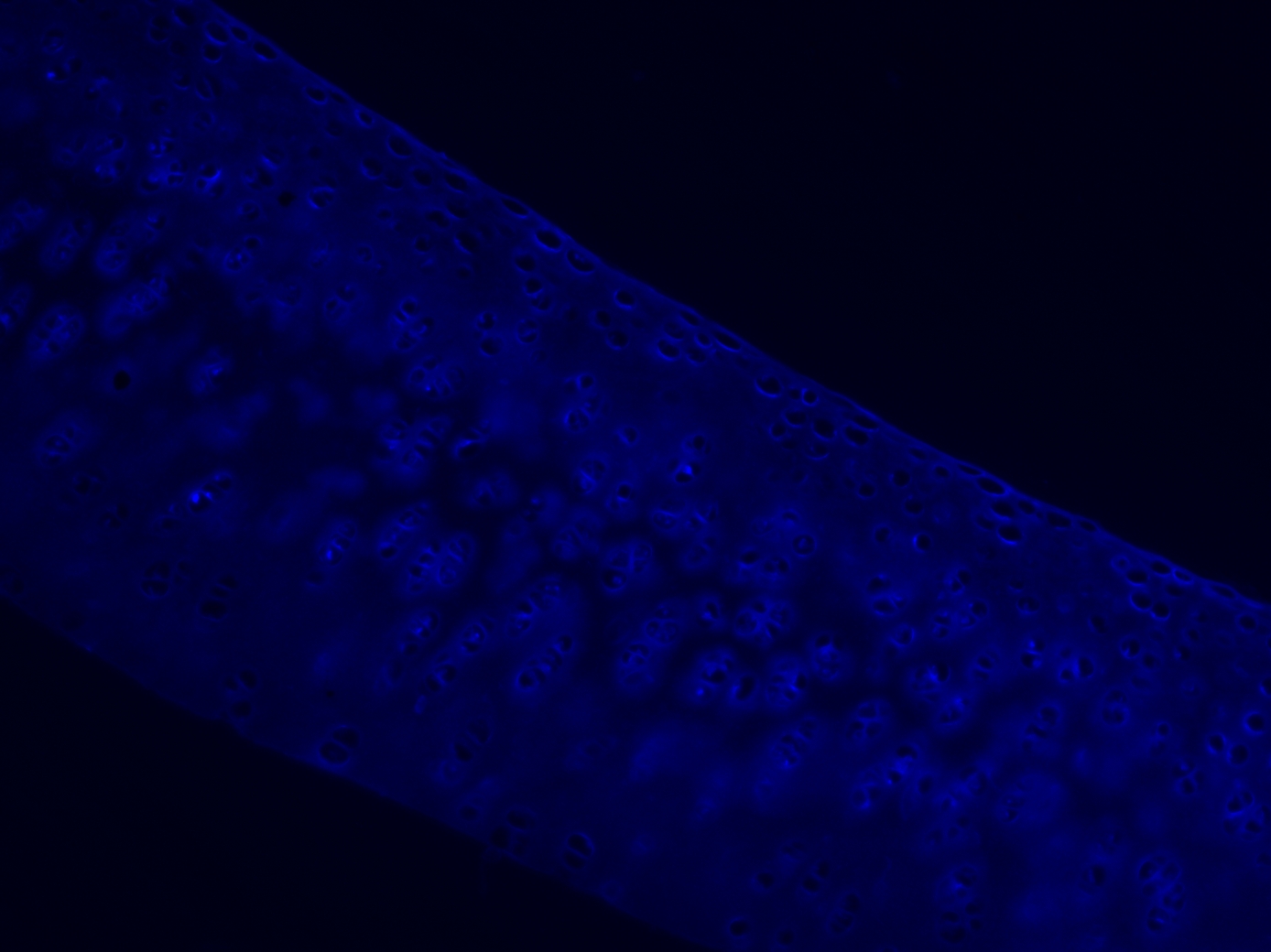

Supplement: Supplemental Information 1 [file peerj-08-8972-s001.zip › Figures/Figure 1A DAPI(After).jpg]

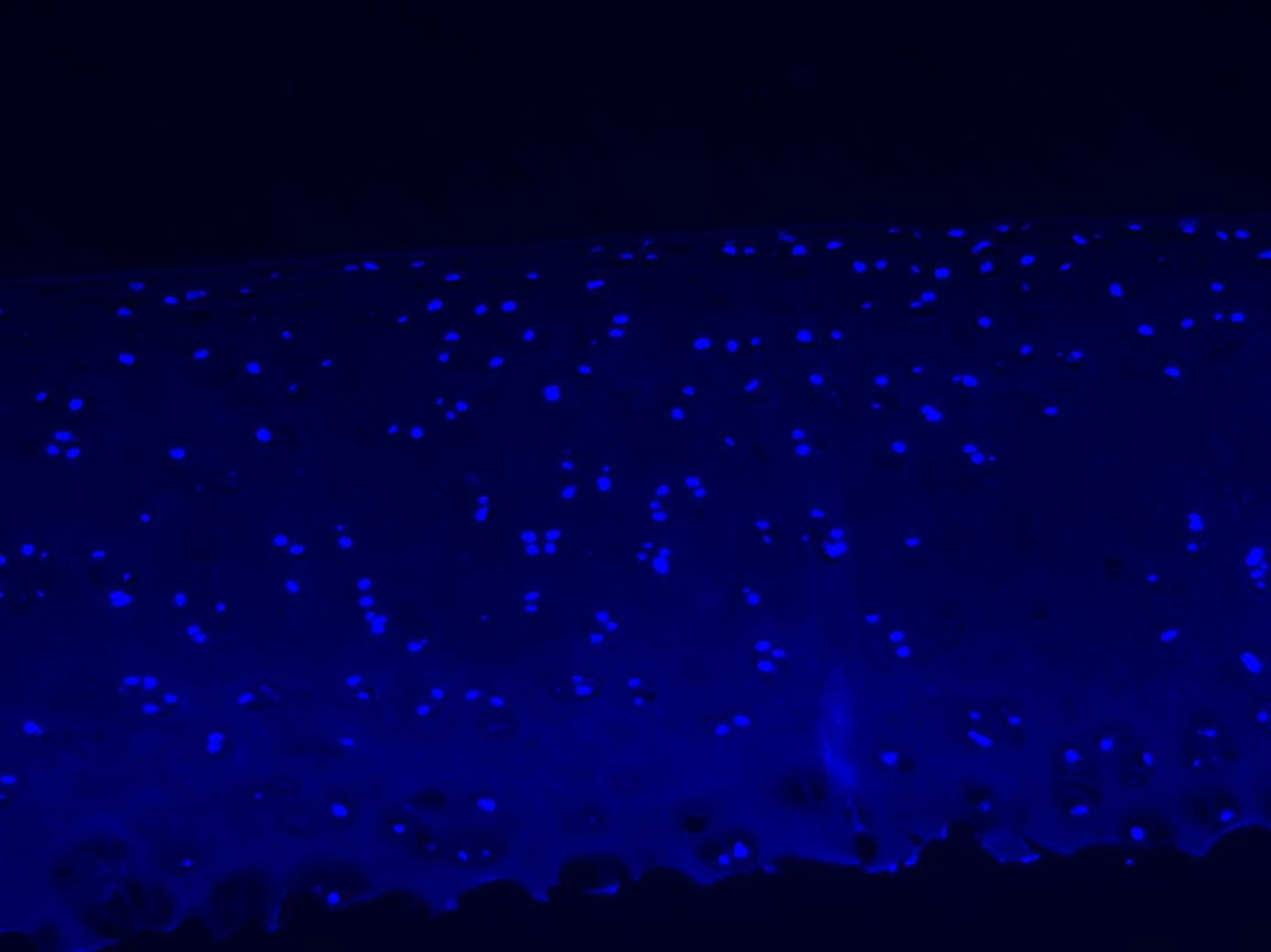

Supplement: Supplemental Information 1 [file peerj-08-8972-s001.zip › Figures/Figure 1A DAPI(Before).jpg]

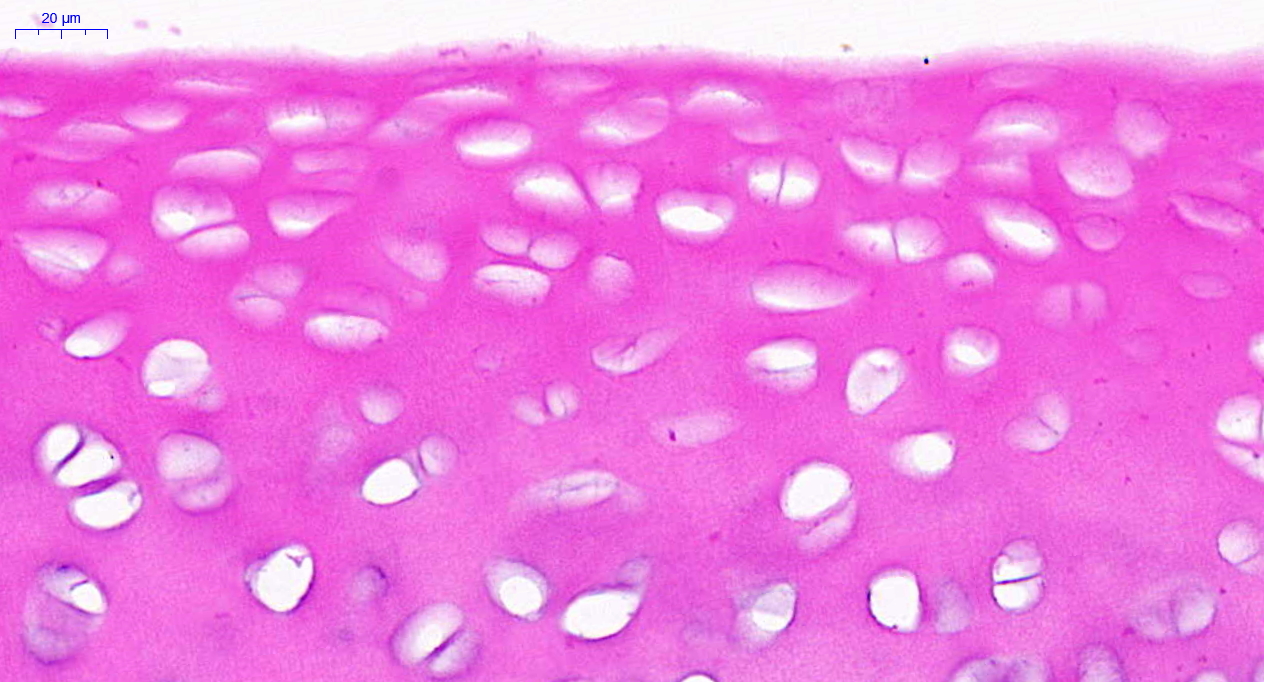

Supplement: Supplemental Information 1 [file peerj-08-8972-s001.zip › Figures/Figure 1A HE(After).jpg]

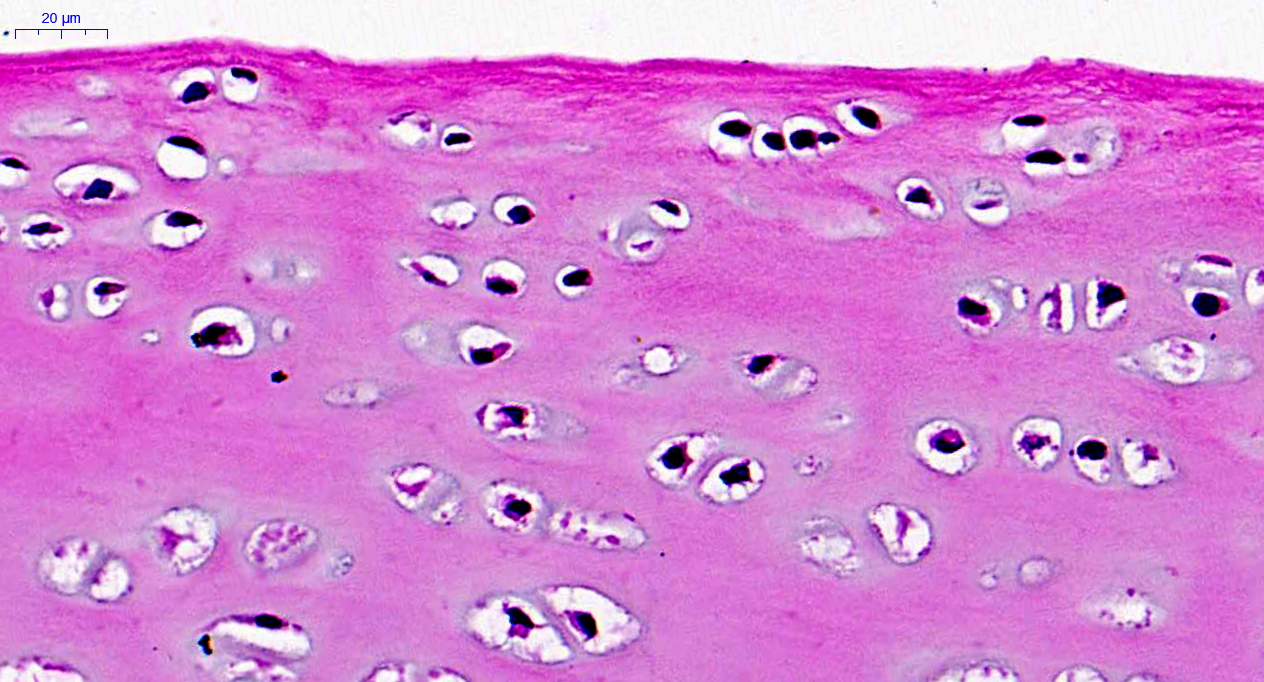

Supplement: Supplemental Information 1 [file peerj-08-8972-s001.zip › Figures/Figure 1A HE(Before).jpg]

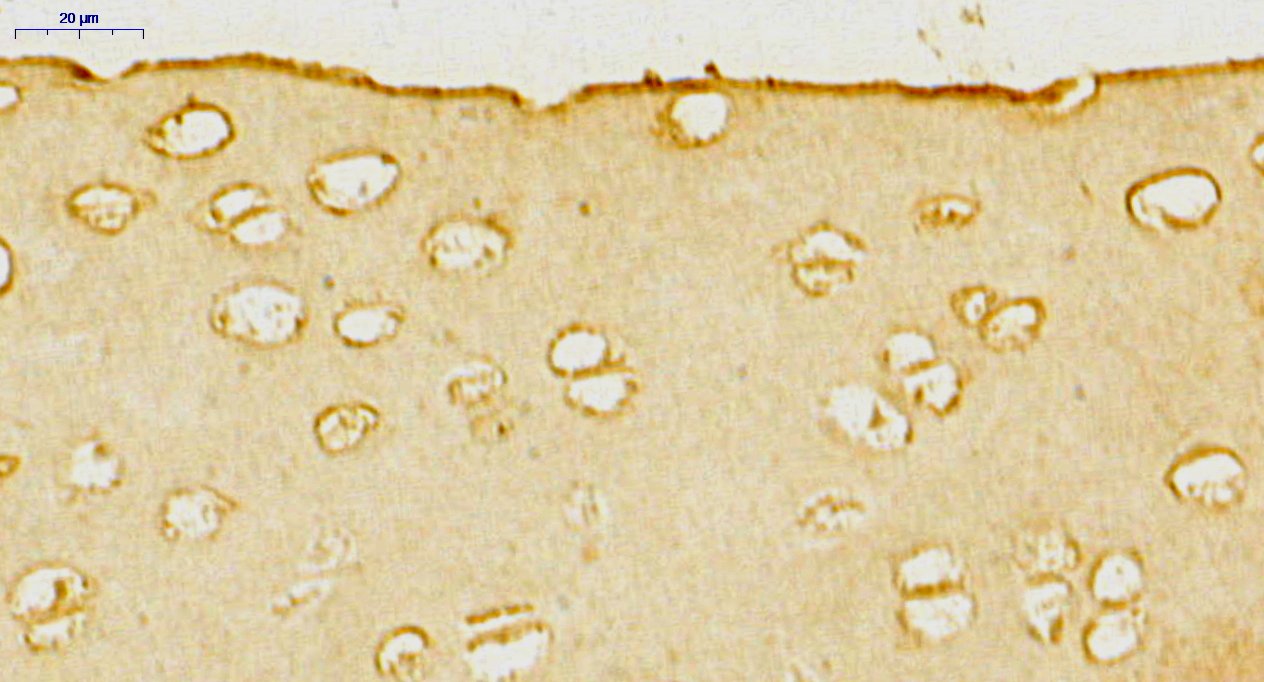

Supplement: Supplemental Information 1 [file peerj-08-8972-s001.zip › Figures/Figure 1A IHC(After).jpg]

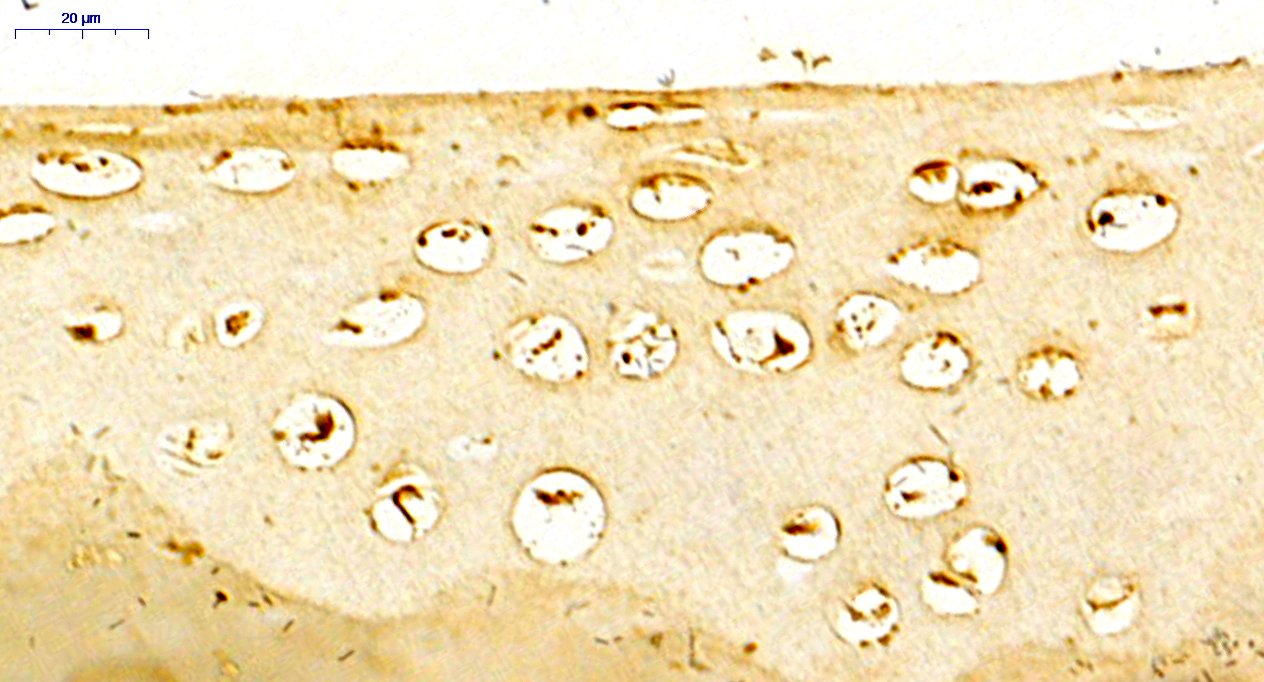

Supplement: Supplemental Information 1 [file peerj-08-8972-s001.zip › Figures/Figure 1A IHC(Before).jpg]

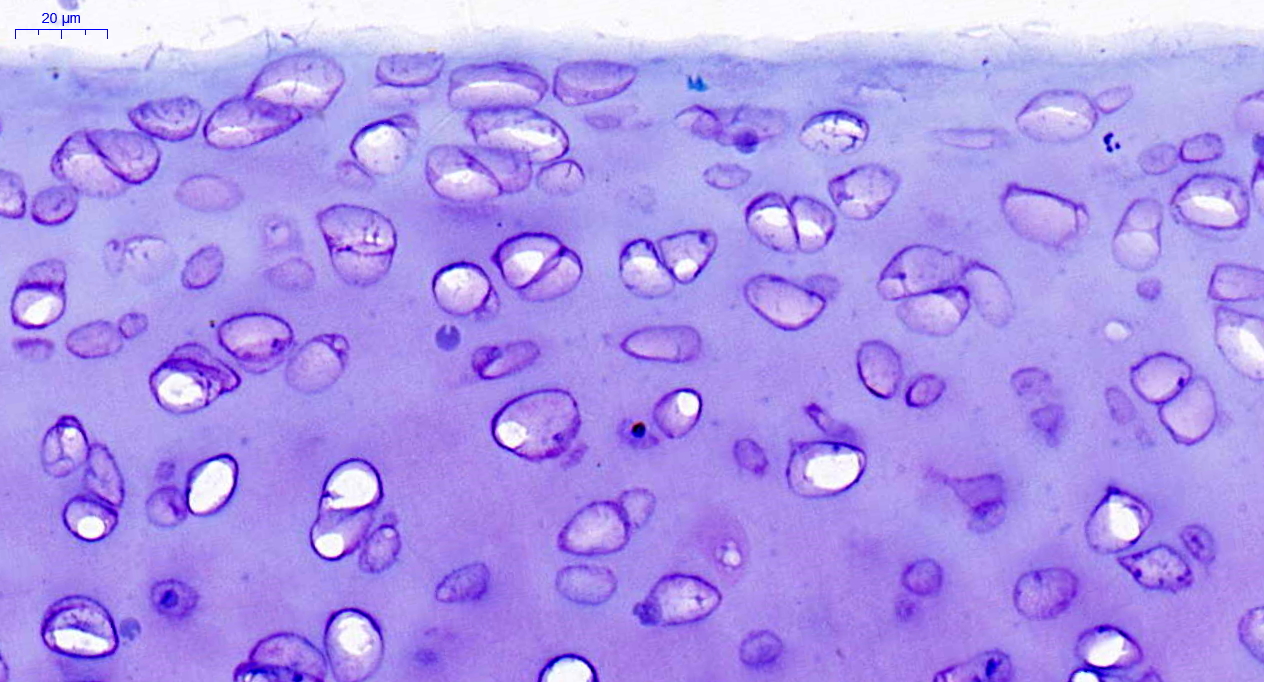

Supplement: Supplemental Information 1 [file peerj-08-8972-s001.zip › Figures/Figure 1A Toluidine Blue Staining(After).jpg]

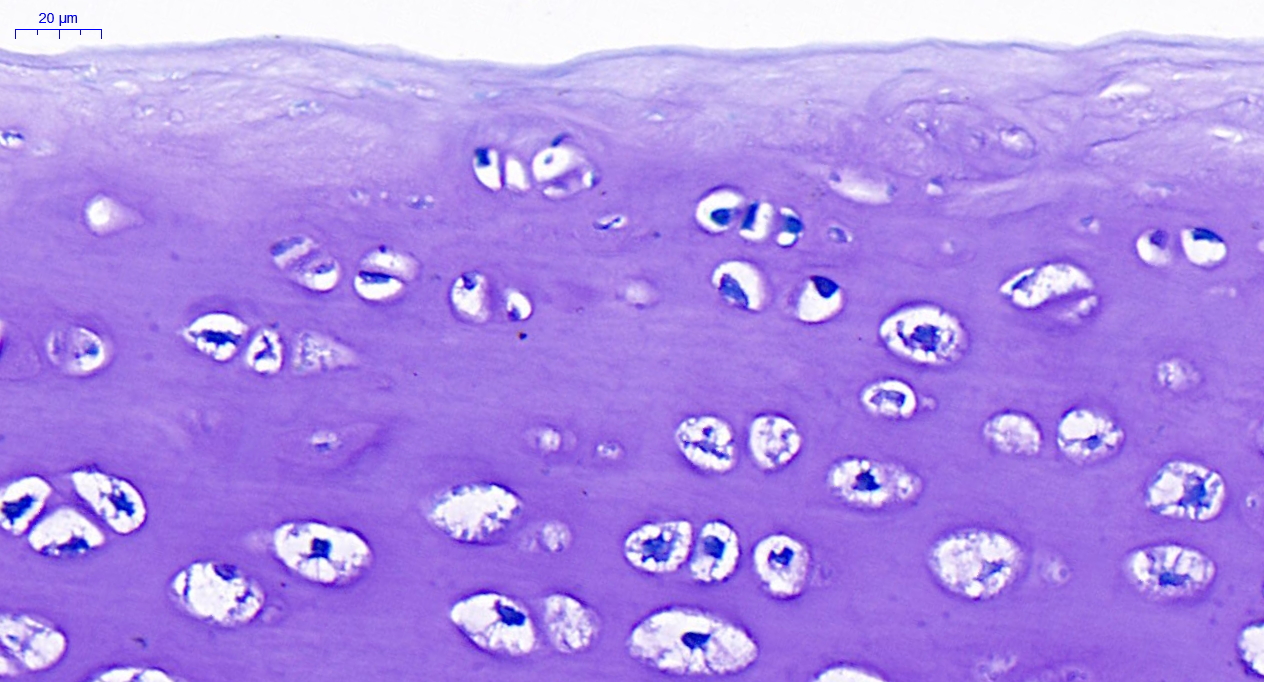

Supplement: Supplemental Information 1 [file peerj-08-8972-s001.zip › Figures/Figure 1A Toluidine Blue Staining(Before).jpg]

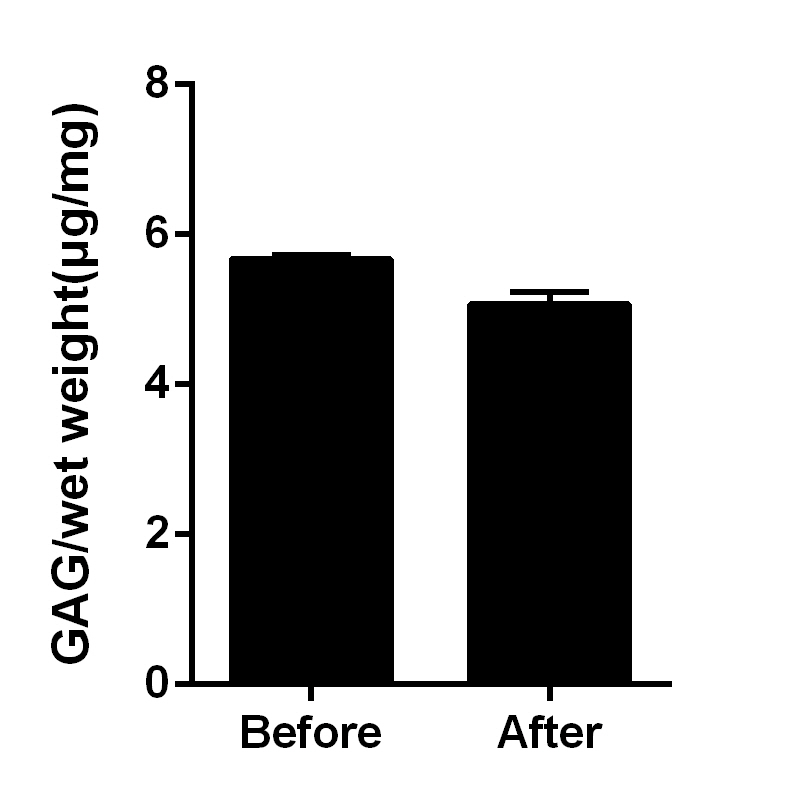

Supplement: Supplemental Information 1 [file peerj-08-8972-s001.zip › Figures/Figure 1B.jpg]

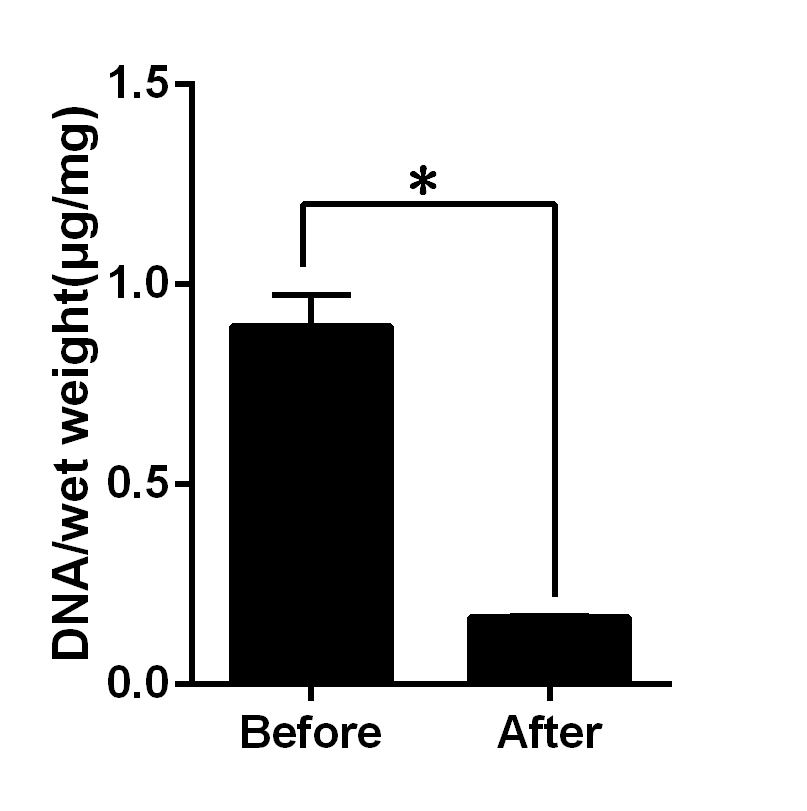

Supplement: Supplemental Information 1 [file peerj-08-8972-s001.zip › Figures/Figure 1C.jpg]

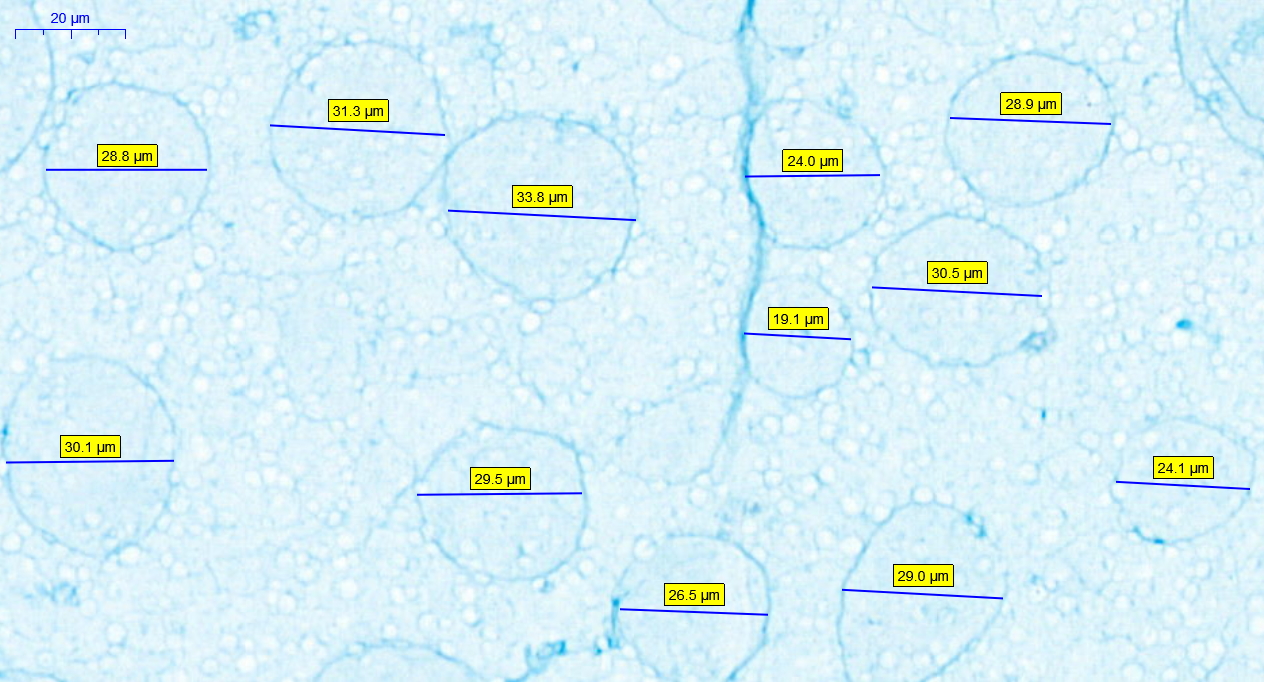

Supplement: Supplemental Information 1 [file peerj-08-8972-s001.zip › Figures/Figure 2 E.jpg]

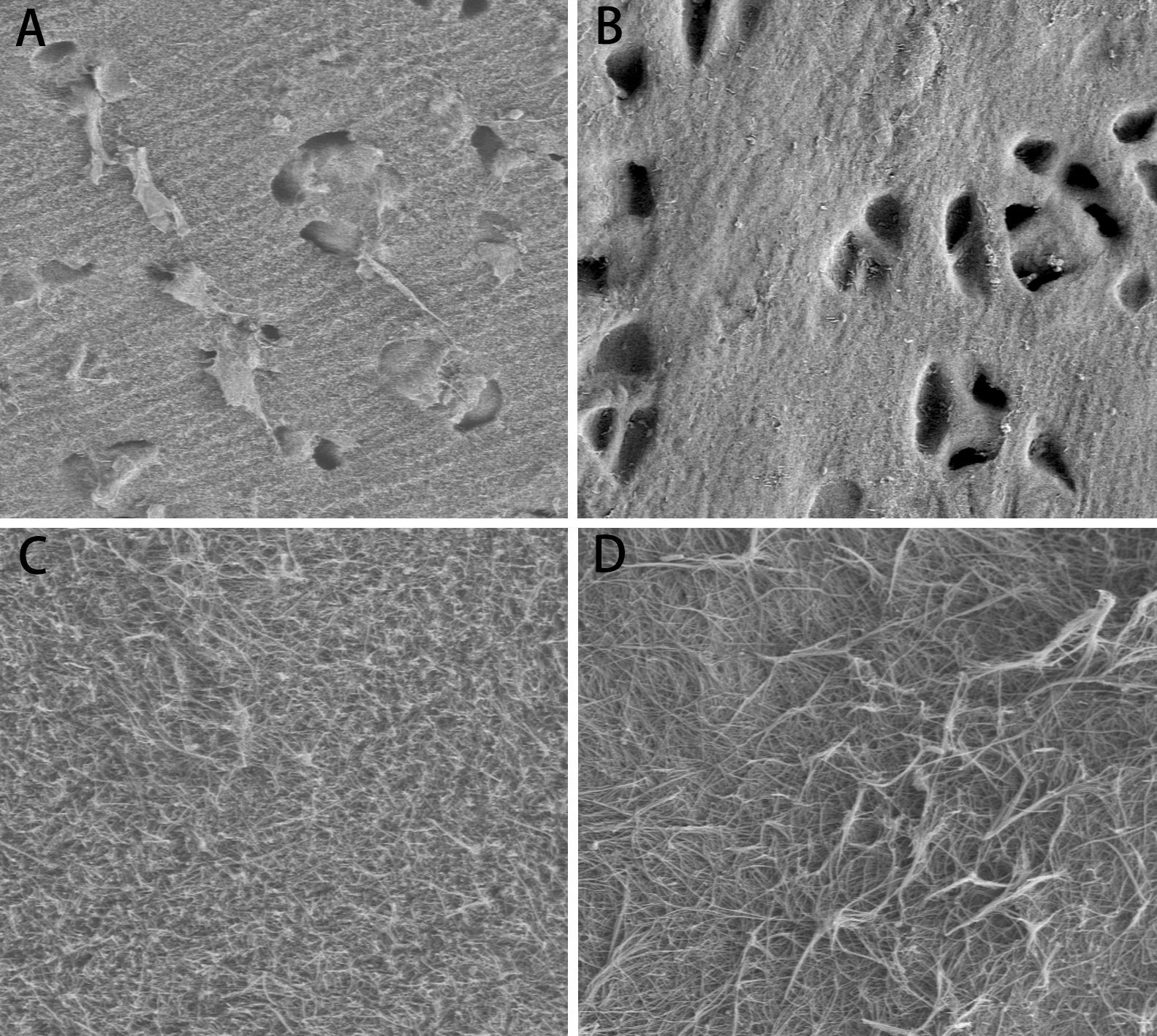

Supplement: Supplemental Information 1 [file peerj-08-8972-s001.zip › Figures/Figure 2 SEM.jpg]

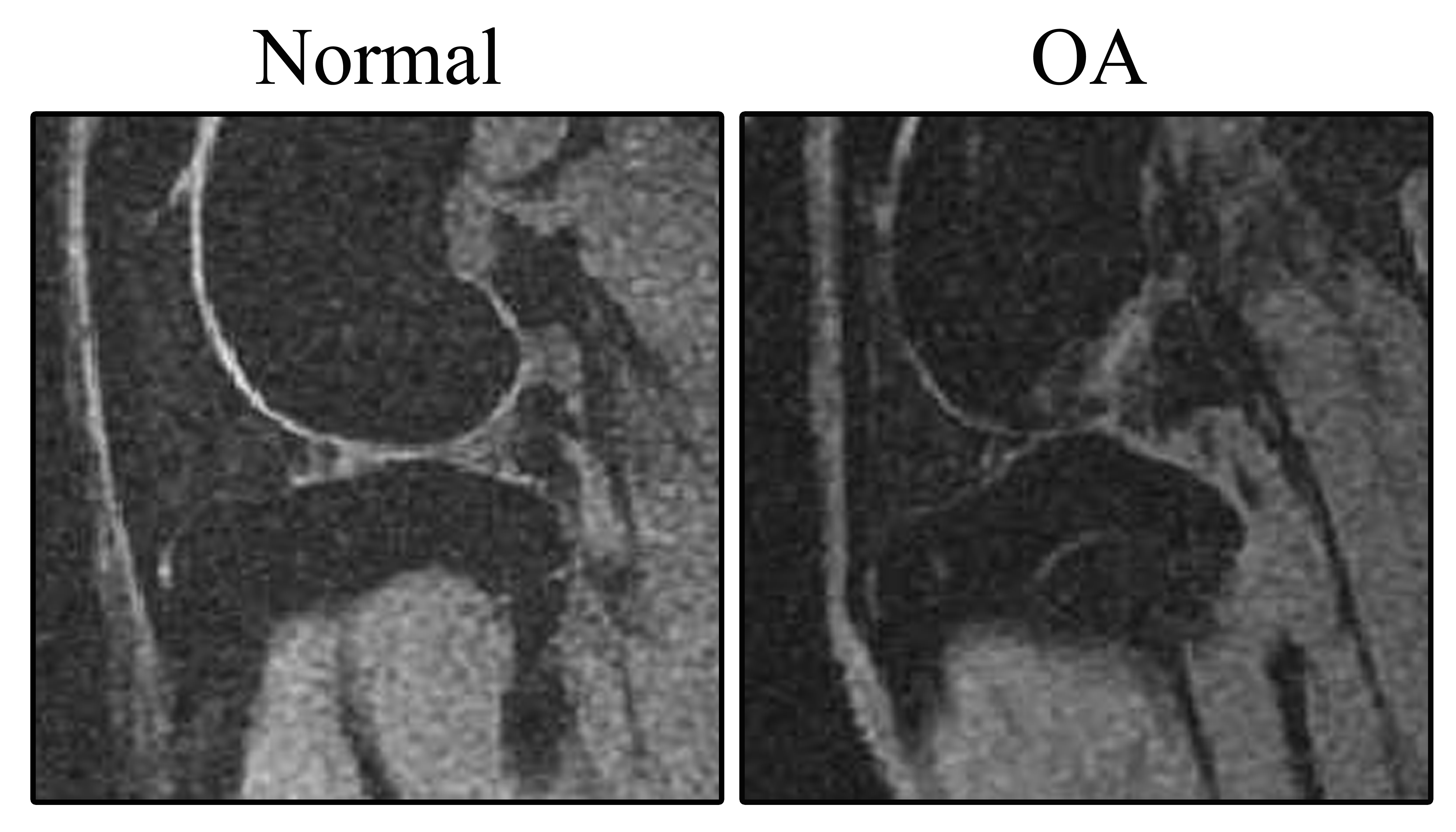

Supplement: Supplemental Information 1 [file peerj-08-8972-s001.zip › Figures/Figure 3 MRI.jpg]

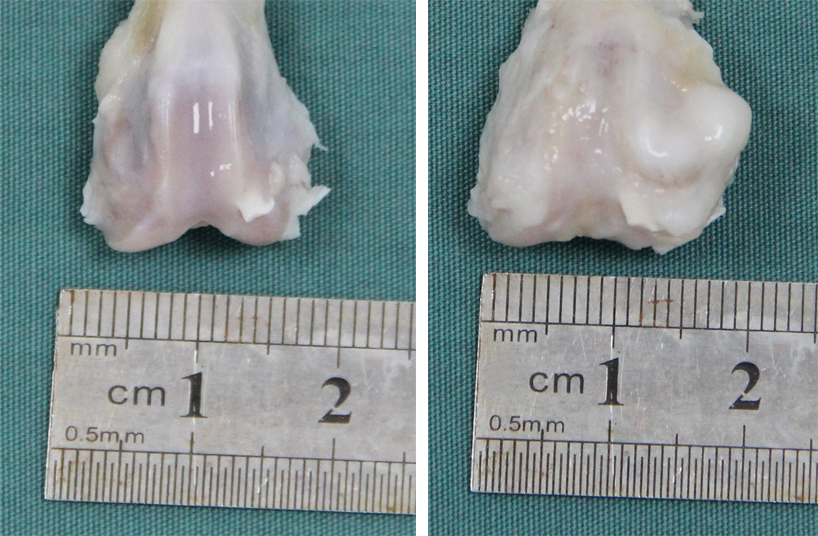

Supplement: Supplemental Information 1 [file peerj-08-8972-s001.zip › Figures/Figure 3.jpg]

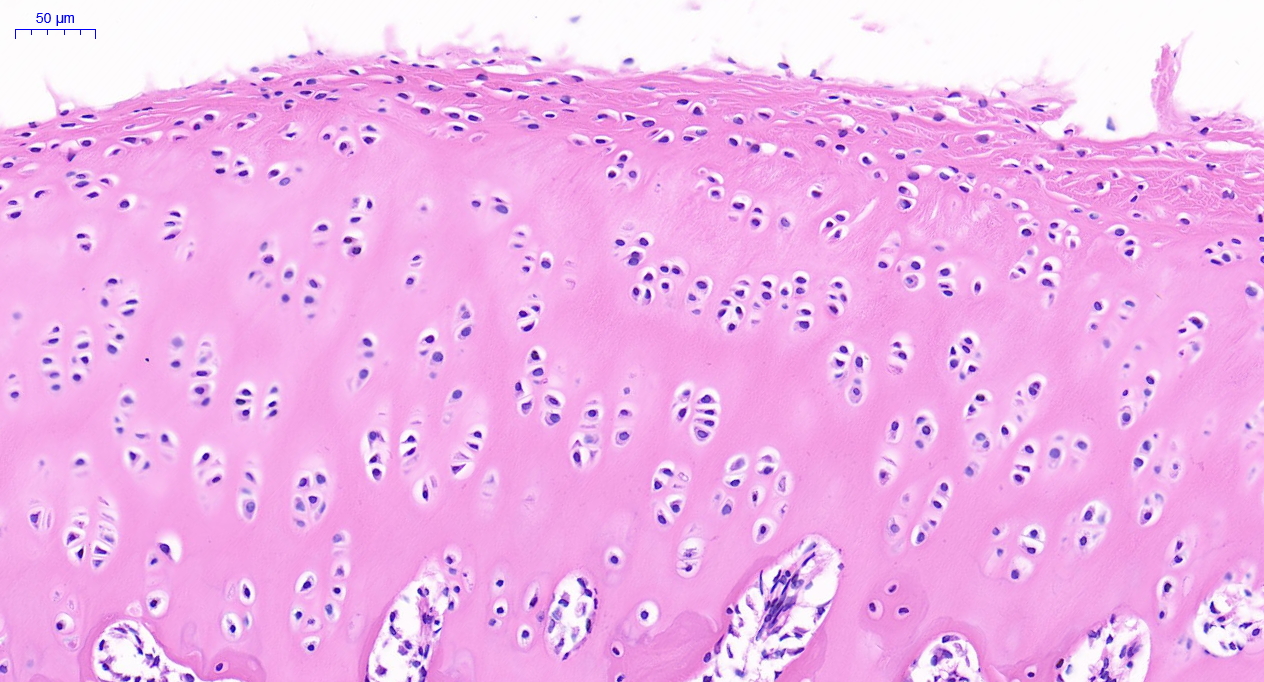

Supplement: Supplemental Information 1 [file peerj-08-8972-s001.zip › Figures/Figure 4 HE1.jpg]

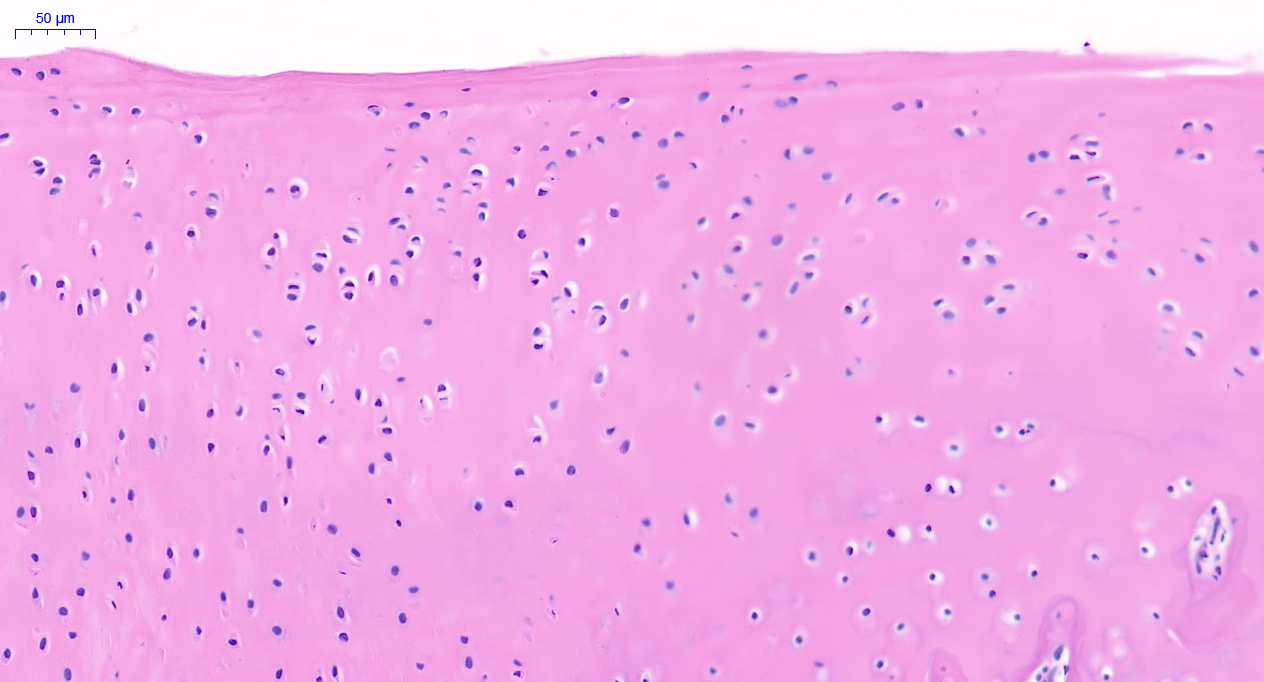

Supplement: Supplemental Information 1 [file peerj-08-8972-s001.zip › Figures/Figure 4 HE2.jpg]

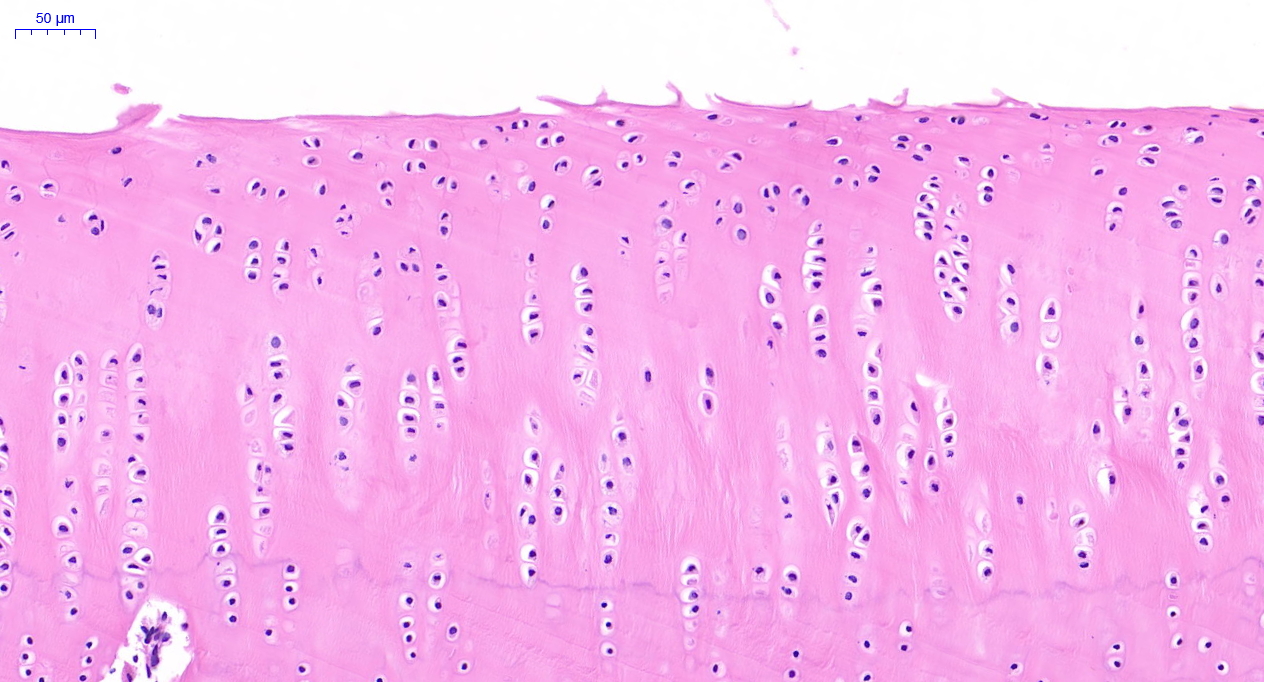

Supplement: Supplemental Information 1 [file peerj-08-8972-s001.zip › Figures/Figure 4 HE3.jpg]

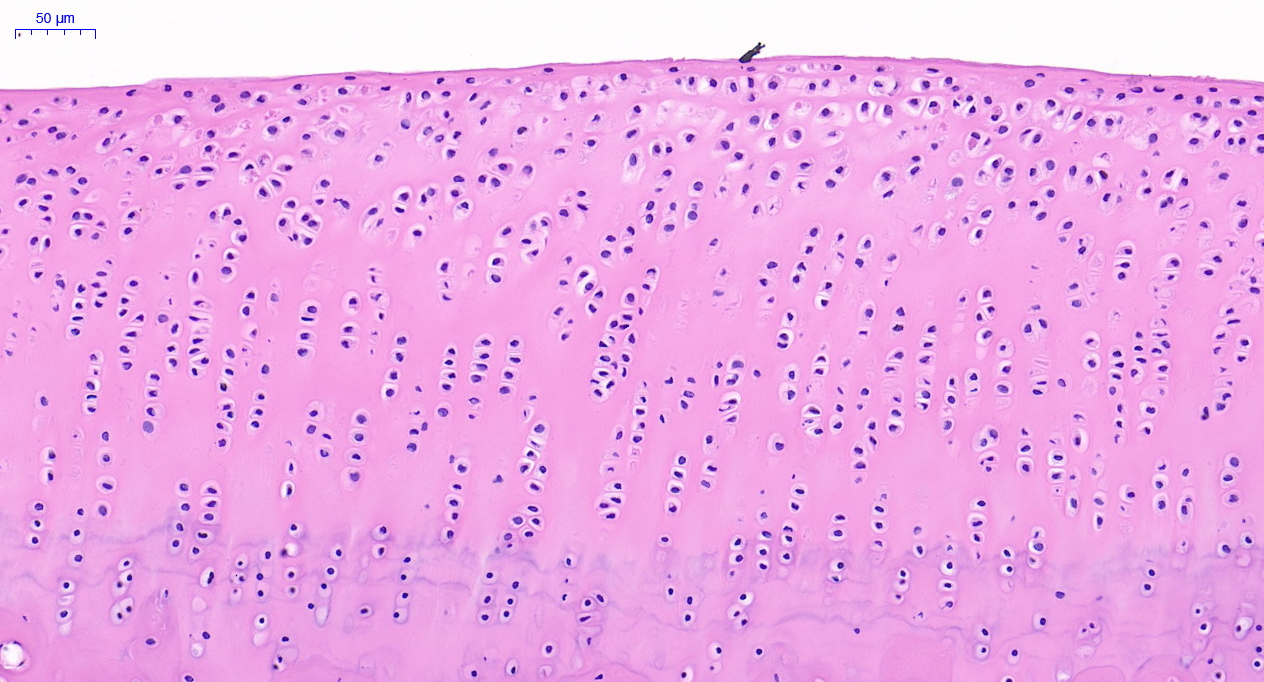

Supplement: Supplemental Information 1 [file peerj-08-8972-s001.zip › Figures/Figure 4 HE4.jpg]

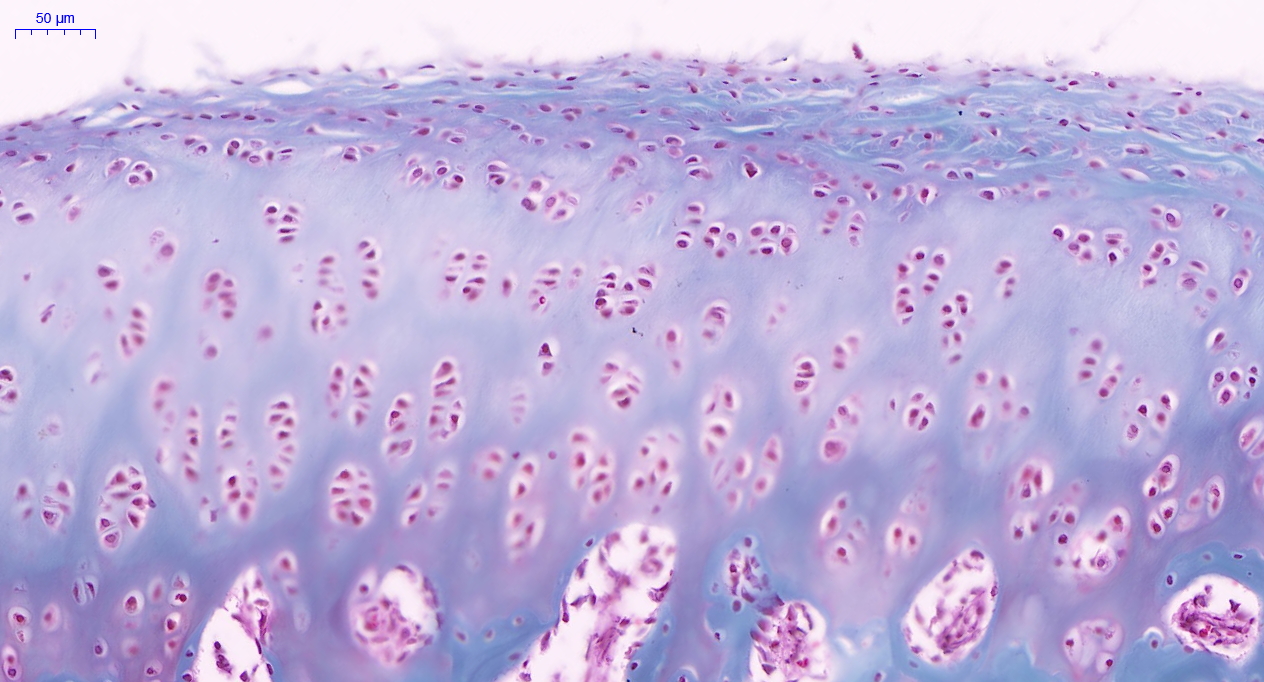

Supplement: Supplemental Information 1 [file peerj-08-8972-s001.zip › Figures/Figure 4 Safranin O-Fast Green Staining 1.jpg]

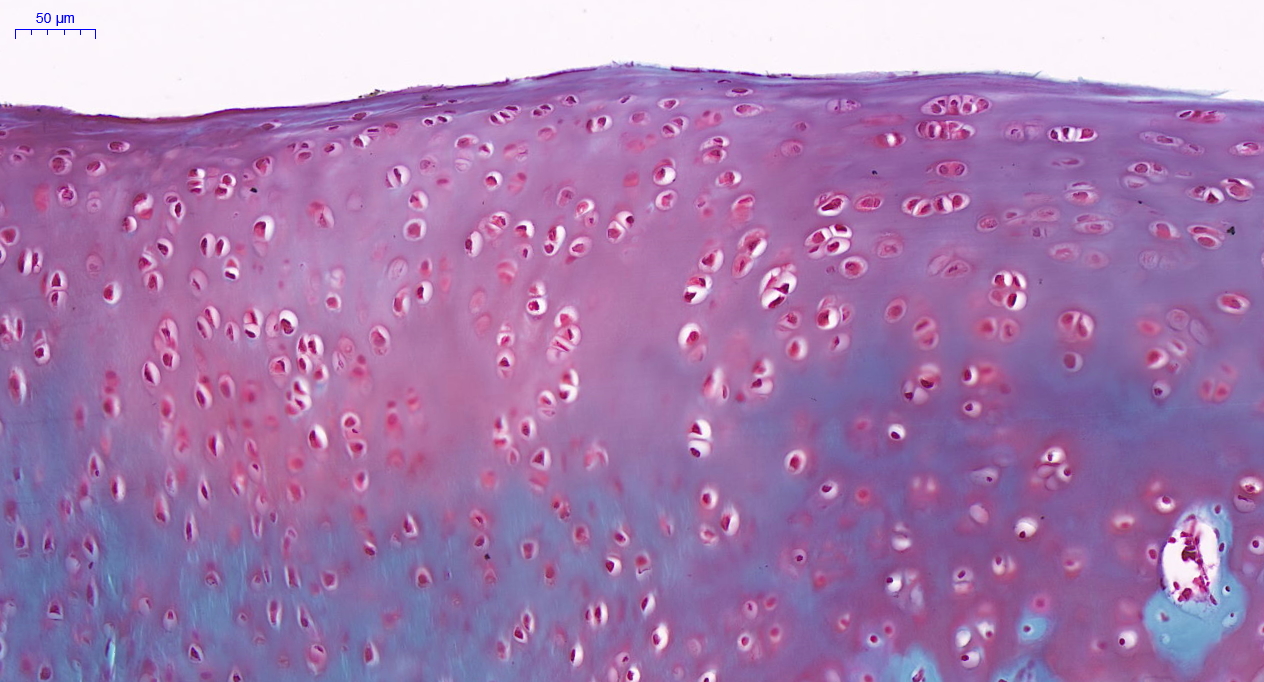

Supplement: Supplemental Information 1 [file peerj-08-8972-s001.zip › Figures/Figure 4 Safranin O-Fast Green Staining 2.jpg]

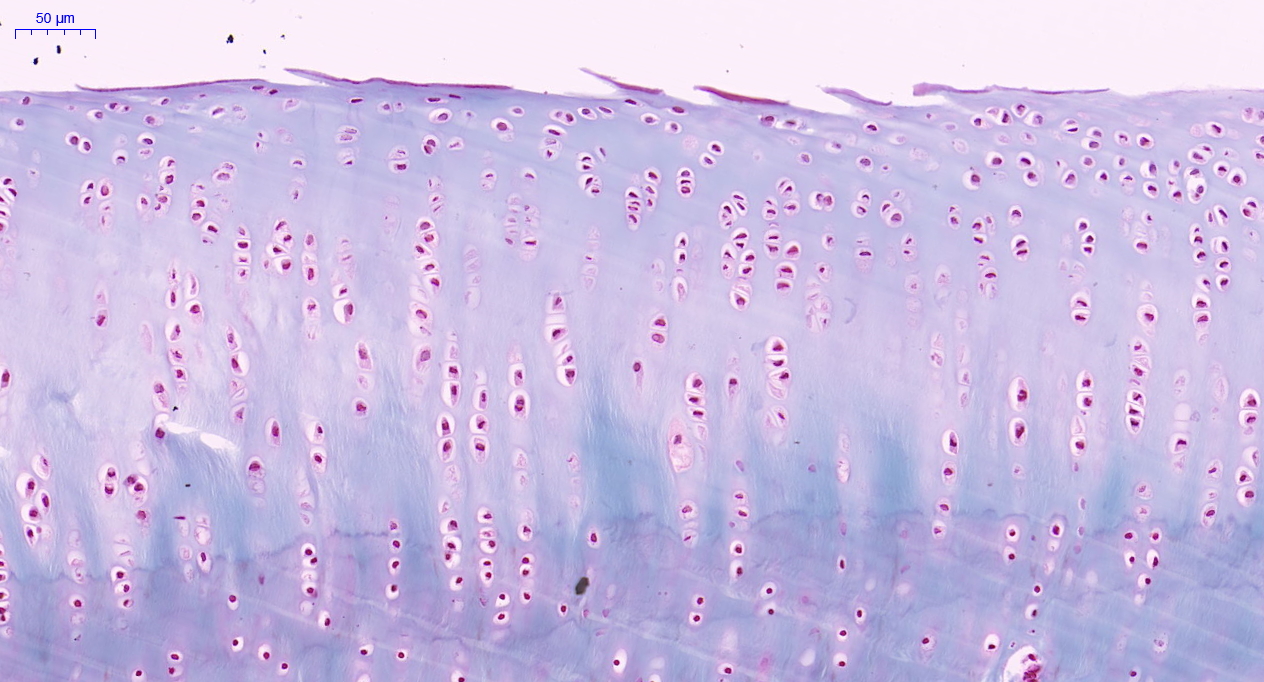

Supplement: Supplemental Information 1 [file peerj-08-8972-s001.zip › Figures/Figure 4 Safranin O-Fast Green Staining 3.jpg]

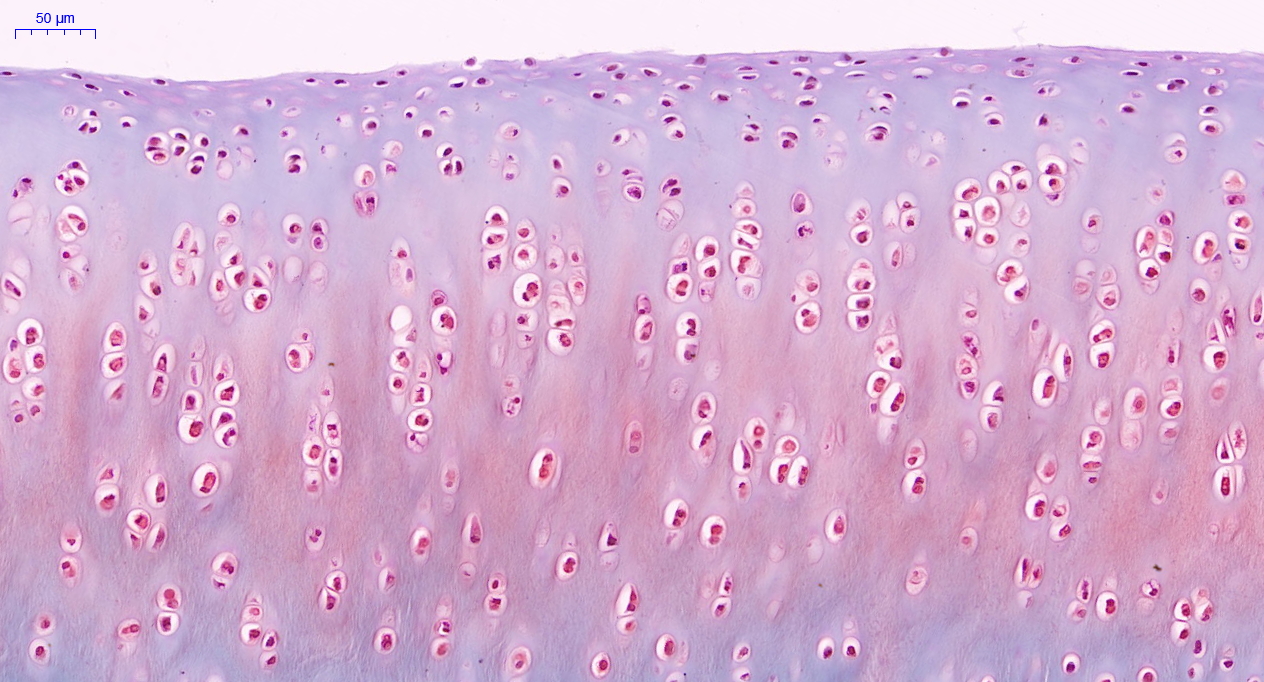

Supplement: Supplemental Information 1 [file peerj-08-8972-s001.zip › Figures/Figure 4 Safranin O-Fast Green Staining 4.jpg]

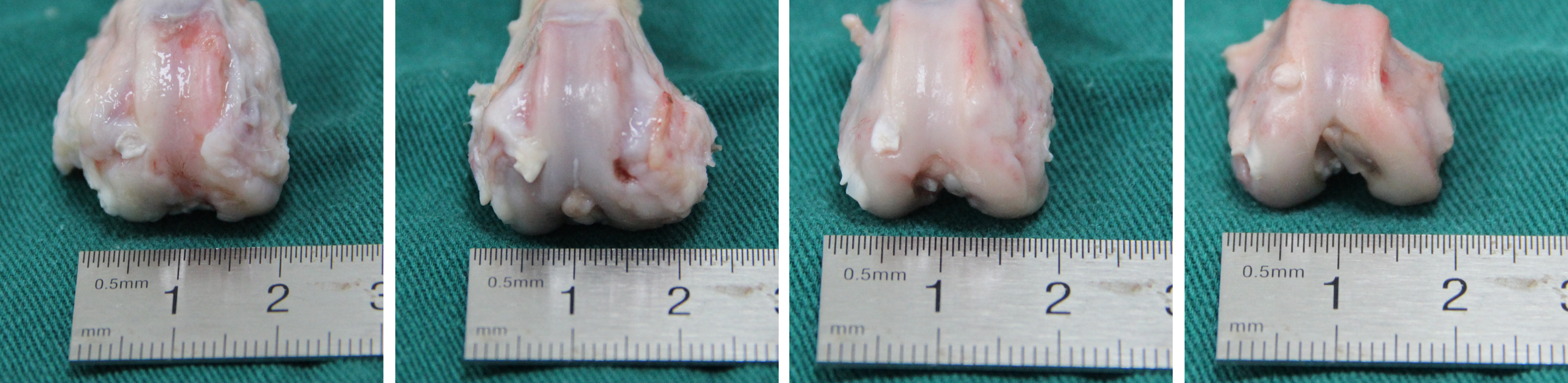

Supplement: Supplemental Information 1 [file peerj-08-8972-s001.zip › Figures/Figure 4.jpg]
